# Circulating cell-free DNA (cfDNA) in patients with medullary thyroid carcinoma is characterized by specific fragmentation and methylation changes with diagnostic value

**DOI:** 10.1186/s40364-023-00522-4

**Published:** 2023-09-19

**Authors:** Anna Citarella, Zein Mersini Besharat, Sofia Trocchianesi, Tanja Milena Autilio, Antonella Verrienti, Giuseppina Catanzaro, Elena Splendiani, Zaira Spinello, Silvia Cantara, Patrizia Zavattari, Eleonora Loi, Cristina Romei, Raffaele Ciampi, Luciano Pezzullo, Maria Grazia Castagna, Antonio Angeloni, Rosella Elisei, Cosimo Durante, Agnese Po, Elisabetta Ferretti

**Affiliations:** 1 Department of Experimental Medicine, Sapienza University of Rome, Rome, 00161 Italy; 2 Department of Translational and Precision Medicine, Sapienza University of Rome, Rome, 00161 Italy; 3 Department of Movement, Human and Health Sciences, University of Foro Italico, 00135 Rome, Italy; 4 Department of Medical, Surgical and Neurological Sciences, University of Siena, Siena, 53100 Italy; 5 Department of Biomedical Sciences, Unit of Biology and Genetics, University of Cagliari, Cagliari, 09042 Italy; 6 Endocrine Unit, Department of Clinical and Experimental Medicine, University of Pisa, Pisa, 56126 Italy; 7 Thyroid Surgical Unit, IRCCS Fondazione G.Pascale, Naples, 80131 Italy; 8 Department of Molecular Medicine, Sapienza University of Rome, Rome, 00161 Italy

**Keywords:** Medullary thyroid carcinoma, Rare tumour, Cell-free DNA, Circulating DNA methylation, Circulating DNA fragmentation, Circulating biomarker

## Abstract

Medullary Thyroid Carcinoma (MTC) is a rare neuroendocrine tumour whose diagnosis includes evaluating calcitonin serum levels, which can present fluctuations unrelated to MTC. Here, we investigated circulating DNA fragmentation and methylation changes as potential biomarkers using ddPCR on cell-free DNA (cfDNA) isolated from the plasma of MTC patients. For cfDNA fragmentation analysis, we investigated the fragment size distribution of a gene family and calculated short fragment fraction (SFF). Methylation analyses evaluated the methylation levels of CG_16698623, a CG dinucleotide in the MGMT gene that we found hypermethylated in MTC tissues by analyzing public databases. The SFF ratio and methylation of CG_16698623 were significantly increased in plasma from MTC patients at diagnosis, and patients with clinical remission or stable disease at follow-up showed no significant SFF difference compared with healthy subjects. Our data support the diagnostic value of cfDNA traits that could enable better management of MTC patients.

**Supplementary Information:**

The online version contains supplementary material available at 10.1186/s40364-023-00522-4.

## To the editor

Medullary thyroid carcinoma (MTC) is a rare tumor arising from parafollicular C-cells [1]. Patients with intrathyroidal tumour have an excellent 10-year survival, which worsens in patients with nodal or distant metastasis at diagnosis [1].

MTC diagnosis includes fine-needle aspiration biopsy (FNAB) in thyroid nodules and deregulated serum and/or FNAB calcitonin (Ct) levels. However, the use of serum Ct presents limitations, such as FNAB being invasive and identifying half of the MTCs [2], rare Ct-negative MTCs [3], and altered levels in non MTC-related conditions like C-cell hyperplasia and other neuroendocrine tumors [4, 5]. Therefore, the identification of additional non-invasive biomarkers is a major challenge for diagnosis and disease monitoring.

Plasma cell-free DNA (cfDNA) is released in the bloodstream by cells and is a promising source for diagnostic and prognostic biomarkers, such as tumor-specific mutations and epigenetic features [5]. Indeed, in MTC patients, cfDNA was previously analyzed to evaluate tumor-specific mutations for minimal residual disease and response to treatment monitoring [6, 7]. Epigenetic features of cfDNA, such as fragmentation and methylation patterns, have been investigated: a high proportion of short cfDNA fragments was reported in cancer patients [8], and circulating methylation signatures were recently exploited for multicancer detection [9]. Of note, cfDNA epigenetic features have never been investigated in MTC.

We therefore evaluated plasma cfDNA fragmentation and methylation using droplet digital PCR (ddPCR) in MTC patients enrolled in the study. Patients’ features and detailed methods can be found in the supplemental material.

**Fig. 1:**
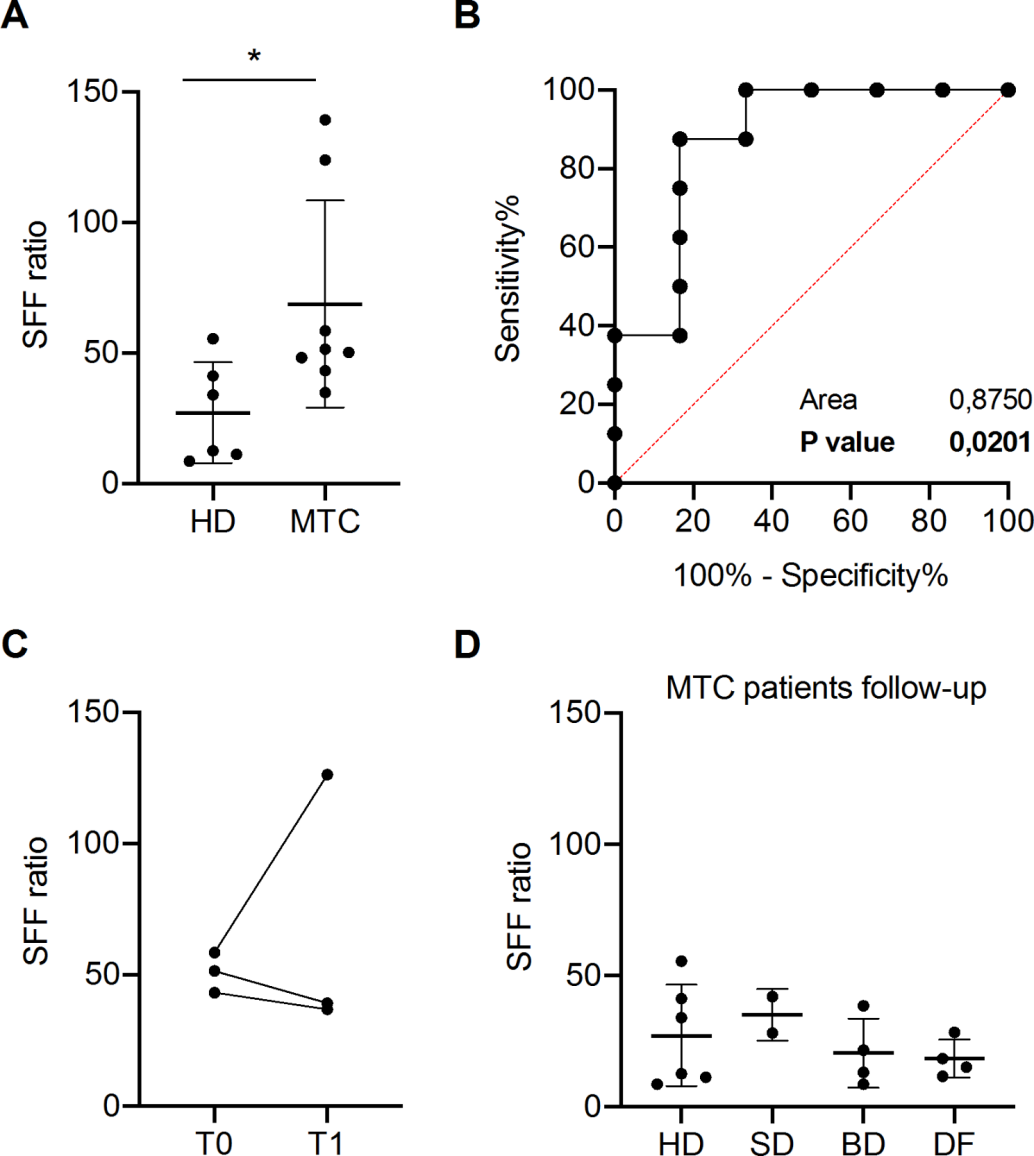
Evaluation of cfDNA fragmentation. **(A)** SFF was calculated as the proportion of short fragments as detailed in supplementary methods in MTC patients and healthy donors (controls, HD) plasma samples. Unpaired t-test * p < 0.05 **(B)** ROC curve of SFF in a cohort of 8 MTC and 6 healthy donors subjects (Area under the curve AUC = 0.8750; p-value = 0.0201). Black line = sensitivity, red line = identity. **(C)** SFF in MTC patient before (T0) and three months after the surgical resection (T1). **(D)** SFF in post-surgery 11 MTC patients and 15 healthy donors (HD) plasma samples. SD (Structural disease); BD (Biochemical disease); DF (Disease Free) compared to HD (Healthy Honors; mean value in black line) SFF.

In fragmentation analysis, we focused on the prevalence of the short fragment fraction (SFF) using an assay that targets three fragment sizes from the conserved Olfactory Receptor (OR) gene family, as previously described [10]. We analyzed pre-surgical samples from 8 MTC patients (Supplementary Table 1) versus 6 controls (Supplementary Table 2) and found that the SFF ratio significantly increased in MTC (Fig. 1A), showing higher levels in patients with extra-thyroid extension (Supplementary Fig. 1A).

Moreover, ROC curve analysis resulted in an area under the curve (AUC) of 0.87 (p = 0.02), showing that the SFF discriminates MTC patients from controls (Fig. 1B). No correlation between the SFF and Ct levels was observed (Supplementary Fig. 1B). SFF in 3 disease-free patients three months after surgery showed no significant decrease compared to pre-surgery SFF levels (Fig. 1C). Notably, the SFF evaluated in a second cohort of 10 post-surgery MTC patients with stable (i.e. structural or biochemical) disease or remission showed no differences compared to healthy donors (Fig. 1D), suggesting that elevated SFF is a feature of patients with active MTC.

To investigate cfDNA methylation, we first searched for alterations by interrogating publicly available methylation data from MTC and normal thyroid (detailed in the supplemental material). Among the top 10 significantly methylated CG dinucleotides in MTC compared to normal thyroid (Supplementary Table 3), we focused on two dinucleotides CG_16698623 and CG_17686260 both located in the MGMT gene since MGMT is hypermethylated in other types of tumors [11]. DdPCR was performed for CG_16698623 (MGMT_623CG) methylation because the flanking sequence of CG_17686260 contained CG dinucleotides with unknown methylation status.

**Fig. 2:**
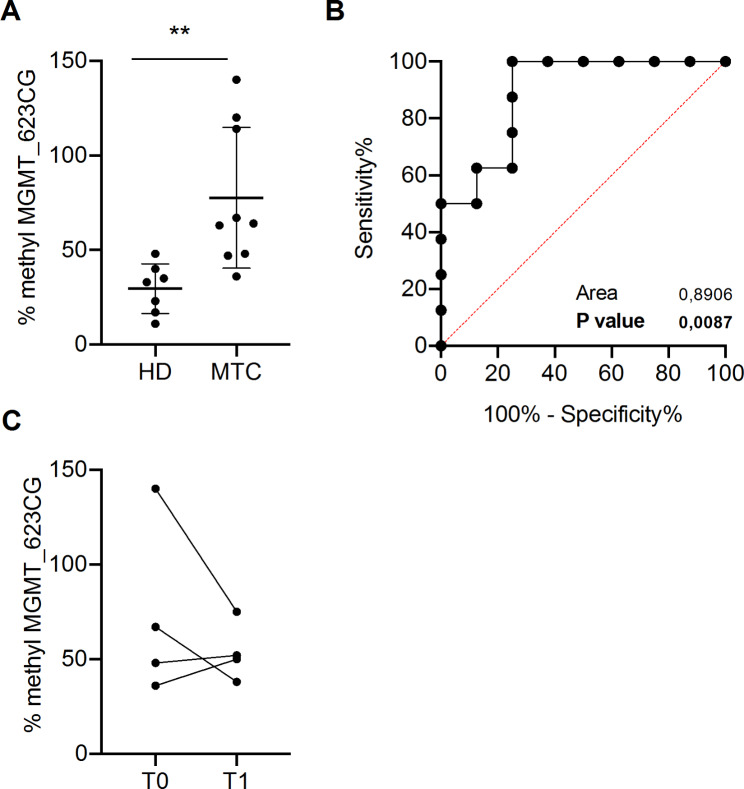
Evaluation of MGMT_623CG methylation. **(A)** MGMT_623CG methylation percentage in MTC patients and healthy donors (HD) plasma samples. The methylation rate was calculated by using the bisulfite converted Human Methylated DNA and human unmethylated DNA as positive and negative controls, respectively. Unpaired t-test * p < 0.05. **(B)** ROC curve of MGMT_623CG methylation percentage in a cohort of 9 MTC patients and 7 healthy donors subjects (AUC = 0.8750; p-value = 0.0201). Black line = sensitivity, red line = identity. **(C)** MGMT_623CG methylation percentage in MTC patients before (T0) and three months after the surgical resection (T1)

We performed the methylation analysis in the pre-surgical plasma of 9 MTC patients compared to controls. MGMT_623CG was hypermethylated in MTC patients (Fig. 2A), with higher levels in patients with extra-thyroid extension (Supplementary Fig. 2A).

The ROC curve analysis with an AUC equal to 0.89 (p = 0.0087) confirmed its performance (Fig. 2B). No significant correlation between MGMT_623CG methylation and Ct levels was observed (Supplementary Fig. 2B). However, two patients with low Ct had high MGMT_623CG methylation, suggesting that MGMT_623CG methylation could identify MTC patients without diagnostic Ct levels.

We evaluated MGMT_623CG methylation in 4 disease-free MTC patients three months after surgical resection, and we observed a negative trend compared to pre-surgery levels (Fig. 2C).

Interestingly, the positive trend in SFF and methylation of MGMT_623CG with advanced tumor stages suggest that these assays could be further investigated for their putative prognostic value. Univariate analyses showed no association of cfDNA features with clinical data such as age and sex (data not shown). The lack of statistical significance for the correlation with staging and early follow-up is presumably due to the small number of samples analyzed, which is the main limitation of our work.

Our data, obtained using a reproducible technology like ddPCR, support the diagnostic value of cfDNA features, which could be clinically valuable as additional non-invasive biomarkers in MTC patients. Validation of our results in independent cohorts could support the clinical application that may facilitate early MTC diagnosis and monitoring. Further studies will likely clarify whether cfDNA features could also guide the extension of surgery in MTC patients.

### Electronic supplementary material

Below is the link to the electronic supplementary material.

Supplementary Material 1

Supplementary Material 2

## Data Availability

All relevant data are included in the manuscript. Methylation data for MTC tissues are available at GSE72729 (https://www.ncbi.nlm.nih.gov/geo/query/acc.cgi?acc=GSM1869223); methylation data for normal thyroid are available at TCGA data https://portal.gdc.cancer.gov/.

## List of abbreviations

MTC: Medullary thyroid carcinoma
Ct: Calcitonin
cfDNA: Cell-free DNA
ddPCR: Droplet Digital PCR
SFF: Short fragment fraction
AUC: area under the curve
