# Supplementary material for: Circulating cell-free DNA (cfDNA) in patients with medullary thyroid carcinoma is characterized by specific fragmentation and methylation changes with diagnostic value
Source: Biomark Res. 2023 Sep 19;11:82. doi: 10.1186/s40364-023-00522-4 (PMC10510276; doi:10.1186/s40364-023-00522-4)
Supplement: Supplementary file 1 — Supplementary Material 1 [file 40364_2023_522_MOESM1_ESM.docx]

**Circulating cell-free DNA (cfDNA) in patients with medullary thyroid carcinoma is characterized by specific fragmentation and methylation changes with diagnostic value**

Anna Citarella et al.

**Supplemental Material**

**MATERIALS AND METHODS**

*Patients’ cohort*

Twenty sporadic MTC patients were enrolled, and clinicopathological features along with the different types of analyses performed are reported in Supplementary Table 1. Ten patients were enrolled at diagnosis, and for four patients, samples after three months from surgery were available. A second cohort of ten patients characterized by absence of active disease were enrolled after surgery and samples were collected during the follow-up, [range=22-1 years, median=10 years]. For this set of patients, disease status at the time of blood sampling was assessed as follows: structural disease (presence of indolent macroscopic lesions), biochemical disease (high Ct levels but no evidence of MTC related lesions) and in remission (no evidence of disease). A cohort of 20 healthy donors was used as control (Supplementary Table 2). Before enrollment, informed written consent was obtained in accordance with the ethical committee guidelines (Protocol: OTC-CBSS-1114, Ethical committee reference: 4940).

*Sample processing*

Blood samples were collected in EDTA tubes. Plasma isolation was performed using serial centrifugations to avoid the carryover of cellular debris. In detail, the first centrifuge was done at 1300 g for 10’ at room temperature (RT) for plasma separation, then plasma was carefully removed without disturbing the buffy coat and transferred into a new tube that was centrifuged at 1200 g for 20’ at RT. The third centrifuge at 10000 g for 30’ at RT was performed to remove any remaining debris. CfDNA from 1 ml of plasma was extracted using the QIAamp MinElute ccfDNA Mini Kit (Cat. n° 55204) according to the manufacturer’s instructions.

*Droplet digital PCR for fragmentation analysis*

The fragmentation assay was performed according to the protocol published by Alcaide et al(suppl ref 1) with minor modifications. Human olfactory receptor genes (OR) and STAT6 primers and probes were manufactured by Integrated DNA Technologies (USA). The system targets a conserved region of seven human olfactory receptor genes and is composed of one reverse primer and three different forward primers to generate short, medium, and long fragments (OR7-S, OR7-M and OR7-L, respectively). STAT6 was used for the estimation of absolute cfDNA concentrations.

Since the quantification of extracted cfDNA using available methods (i.e. Nanodrop, Qbit) was often unreliable, we performed experiments using cfDNA derived from the standard volume of 1 ml plasma as starting matrix. Fragmentation analysis was performed using the Droplet Digital PCR (ddPCR). ddPCR reactions were set up in a final volume of 22μl and the mixes were prepared using 11μl of the ddPCR Supermix for probes (no dUTP) (Bio-Rad, 1863024), 2μl of cfDNA samples and the primers and probes mix, whose sequence and final concentration can be found in Supplementary table 4. For the detection of short fragments Rv + Fw1 + Short-FAM were used; for the detection of medium fragments Rv + Fw2 + Short-FAM + Medium-FAM were used; for the detection of long fragments Rv + Fw3 + Short-FAM + Medium-FAM + Long-HEX were used.

The PCR mixes for each sample were loaded in a disposable cartridge (Bio-Rad) together with 70 μl of droplet generation Oil (Bio-Rad) and loaded in the QX200 droplet generator (Bio-Rad). 40 μl of droplets were then transferred in a 96-well plate and an endpoint PCR was performed using the following conditions: 95°C for 10 minutes, then 50 cycles of 94°C for 30 seconds and 58°C for 90 seconds, and a final step at 98°C for 10 minutes. Then, the 96 well plate was run in the QX200 Droplet Reader and the quantification of positive droplets was performed using the QuantaSoft software (Bio-Rad). The short fragment fraction (SFF) ratio was calculated as follows: *SFF = (OR7-S/STAT6)/(LFF),* *LFF = (OR7-L/OR7-M)/STAT6*.

*In silico methylation analysis*

Publicly available tissue methylation data of 34 sporadic MTC patients (GSE72729) (suppl ref 2) and 56 normal thyroid samples (TCGA data were downloaded through the GDC Data Portal https://portal.gdc.cancer.gov/) were used to compare methylation levels in MTC and normal thyroid. Data from MTC were generated using the Illumina Infinium 27k Human Methylation Beadchip v1.2, while normal thyroid was analyzed using the Illumina Infinium Human Methylation 450 K BeadChip; therefore, we analyzed loci interrogated by both sources. Beta values were calculated as the ratio of “methylated/methylated plus unmethylated” for CG dinucleotides available in all samples, corresponding to 0 when unmethylated and 1 when fully methylated. Differences between groups were assessed by performing multiple t-tests without assuming a consistent SD and applying the correction for multiple comparisons using the Holm-Sidak Method in GraphPad Prism software Version 6 (San Diego, California, USA).

*cfDNA methylation analysis*

We performed bisulfite conversion of cfDNA extracted from 500 ul of plasma, using the EZ DNA Methylation kit (Cat. N° 5002, ZYMO RESEARCH) according to the manufacturer’s instructions.

The methylation analysis was performed by analyzing the MGMT CG_16698623 region, using ddPCR. The primer pair was designed to encompass the GC dinucleotide of interest, and the probe was designed to specifically bind the methylated form of the dinucleotide (Supplementary table 5). CfDNA input was normalized through the 4PLEX system of primers and probes as previously described (suppl ref 1) and quantification was performed using the PodCall package (Suppl ref 3). Primers and probes for the MGMT CG_16698623 amplicon are listed in Supplementary table 5. The methylation rate was calculated by using the positive control Human Methylated DNA (EpiTect PCR Control DNA Set, Cat. n° 59695), while the specificity of the designed primers and probe was verified using the bisulfite converted Human Unmethylated DNA (EpiTect PCR Control DNA Set, Cat. n° 59695). ddPCR was performed as described for fragmentation analysis, using 2 ul of bisulfite-converted DNA as input.

*Statistical analysis*

Statistical analyses were performed using the GraphPad Prism software Version 6 (San Diego, California, USA). Differences between groups were evaluated using the Mann–Whitney test or one-way ANOVA test, where appropriate. Receiver operating characteristic (ROC) curves were also performed using GraphPad Prism version 6 (San Diego, California, USA) and the Area under the ROC curve was calculated.

Univariate analyses were performed in IBM SPSS Statistics version 27 (Armonk, New York, USA), using the General Linear Model (GLM) for the two aspects of interest distinctly, cfDNA Methylation and cfDNA fragmentation. In detail, the cfDNA Methylation ddPCR values were used as a dependent variable and other clinical features (age, sex and age in two categories [1: over 50 years old and 0: under 50 years old]) as fixed factors. Similarly, for cfDNA fragmentation the SFF ratio ddPCR data was used as a dependent variable and other clinical features (age, sex and age in two categories [1: over 50 years old and 0: under 50 years old]) as fixed factors. Univariate analyses were performed to determine the significance of the clinical features on the cfDNA methylation and fragmentation. The Parameter Estimates summarized the effect of each clinical feature for each aspect of interest, cfDNA methylation and cfDNA fragmentation. P-values of less than 0.05 were considered as statistically significant.

**SUPPLEMENTARY TABLES LEGENDS**

**Supplementary Table 1.** Features of MTC patients enrolled for the methylation and fragmentation analysis.

**Supplementary Table 2.** Features of Healthy Donors (HD, controls) enrolled for the methylation and fragmentation analysis.

**Supplementary Table 3.** List of top 10 CG dinucleotides hypermethylated in MTC tissues versus 56 normal thyroid.

**Supplementary Table 4.** List of Primers, probes and concentrations used for fragmentation analysis.

**Supplementary Table 5.** List of Primers and probe used for methylation analysis.

**SUPPLEMENTARY FIGURE LEGENDS**

**Supplementary Figure 1 A.** SFF in MTC patients according to intrathyroid (TnN0M0) versus extrathyroid extension (TnN1M0+TnN0M1) of disease and healthy donors (HD) plasma samples (mean value in black line) **B.** correlation analysis of SFF ratio and Ct values.

**Supplementary Figure 2 A.** MGMT_623CG methylation percentage in MTC patients according to intrathyroid (TnN0M0) versus extrathyroid extension (TnN1M0+TnN0M1) of disease and healthy donors (HD) plasma samples (mean value in black line) **B.** correlation analysis of MGMT_623CG methylation percentage and Ct values.

**Supplementary reference:**

1. Alcaide M, Cheung M, Hillman J, Rassekh SR, Deyell RJ, Batist G, et al. Evaluating the quantity, quality and size distribution of cell-free DNA by multiplex droplet digital PCR. Sci Reports 2020 101 [Internet]. 2020 Jul 28 [cited 2023 Mar 30];10(1):1–10. Available from: https://www.nature.com/articles/s41598-020-69432-x

2. Mancikova V, Montero-Conde C, Perales-Paton J, Fernandez A, Santacana M, Jodkowska K, et al. Multilayer OMIC Data in medullary thyroid carcinoma identifies the STAT3 pathway as a potential therapeutic target in RETM918T Tumors. Clin Cancer Res. 2017;23(5).

3. Bioconductor - PoDCall [Internet]. [cited 2023 Sep 1]. Available from: https://bioconductor.org/packages/release/bioc/html/PoDCall.html
