# Supplementary material for: Circulating cell-free DNA (cfDNA) in patients with medullary thyroid carcinoma is characterized by specific fragmentation and methylation changes with diagnostic value
Source: Biomark Res. 2023 Sep 19;11:82. doi: 10.1186/s40364-023-00522-4 (PMC10510276; doi:10.1186/s40364-023-00522-4)
Supplement: Supplementary file 2 — Supplementary Material 2 [file 40364_2023_522_MOESM2_ESM.pptx]

## Slide 1
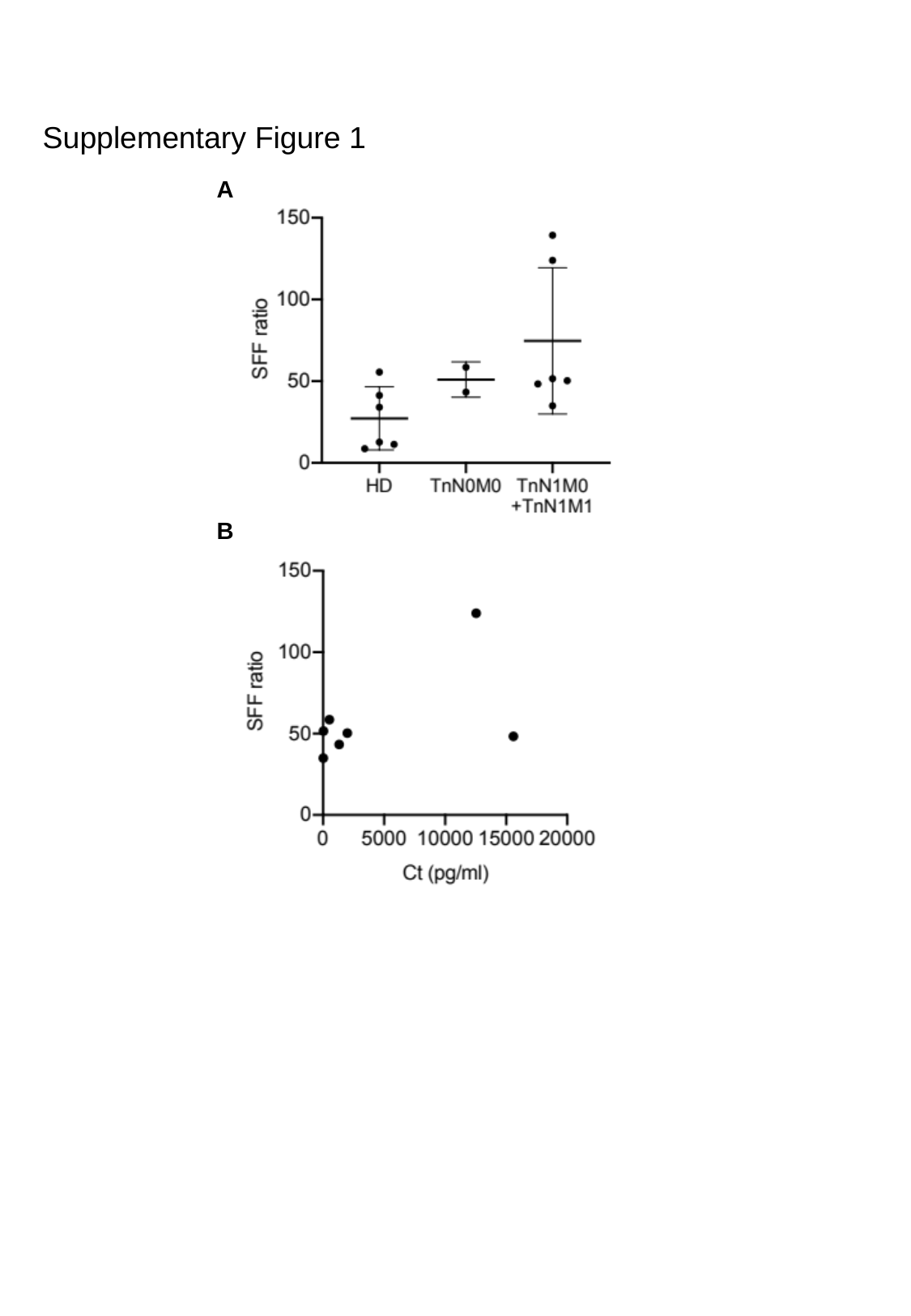

Supplementary Figure 1
A
B

## Slide 2
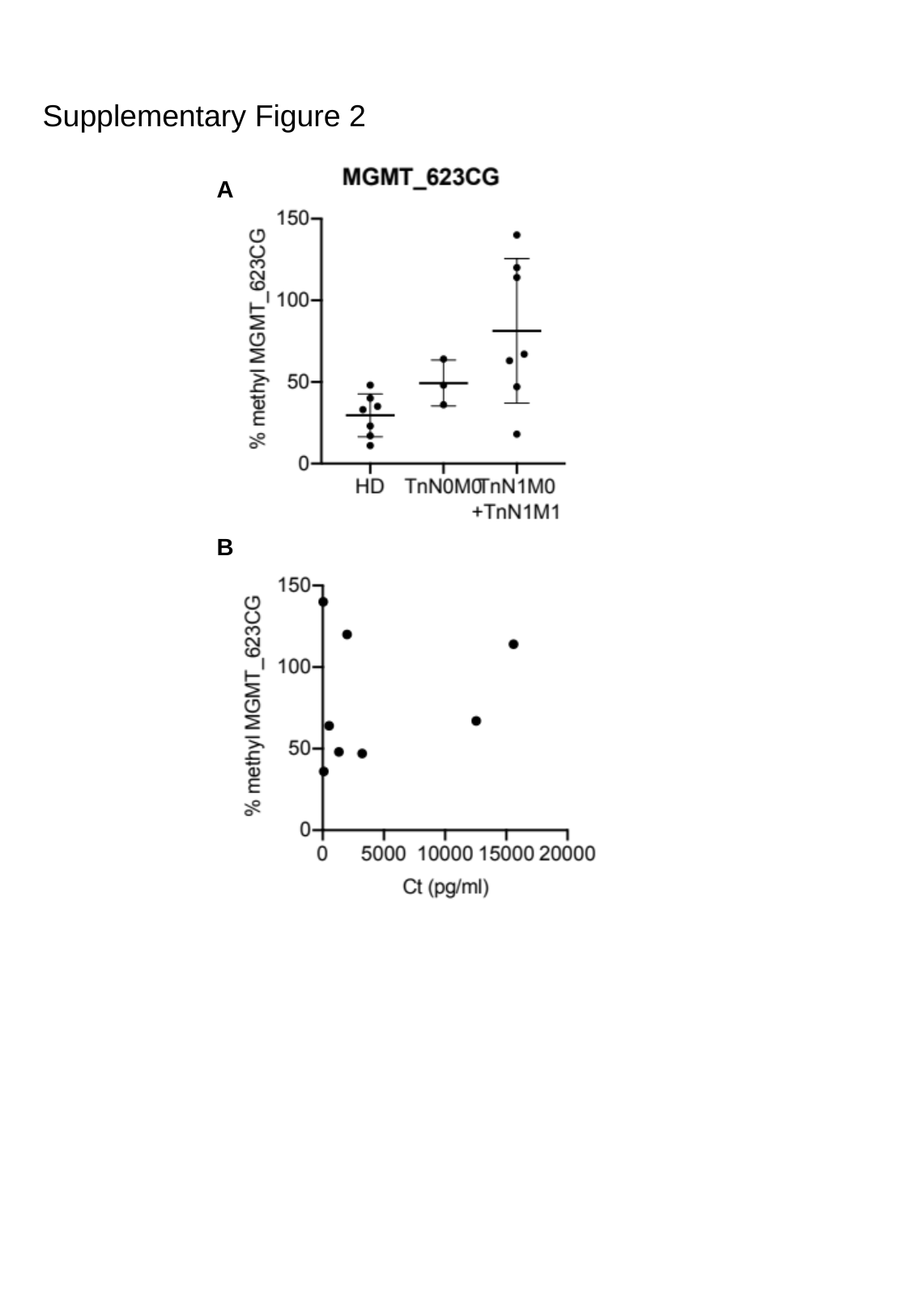

Supplementary Figure 2
A
B

## Slide 3
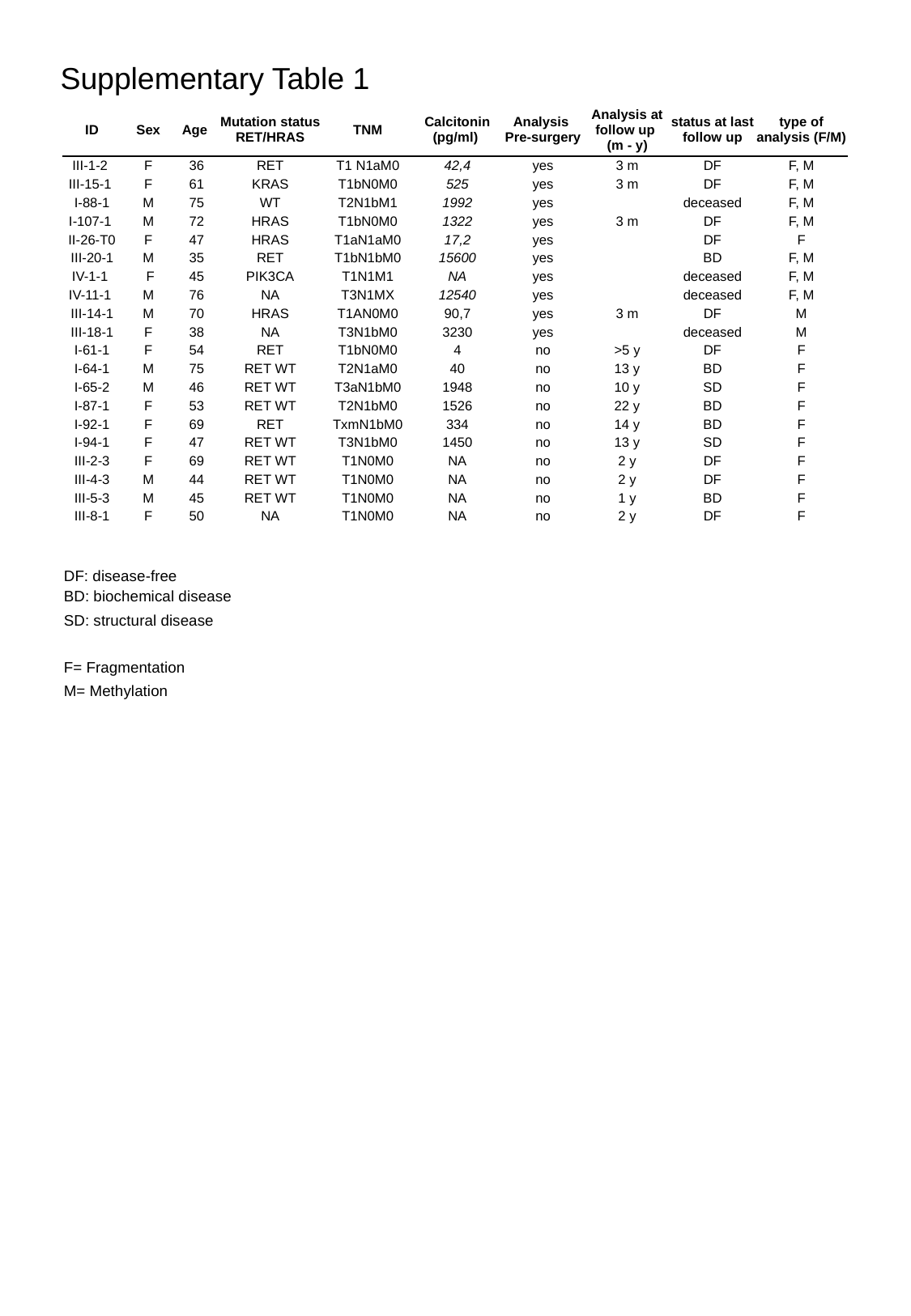

Supplementary Table 1
| ID | Sex | Age | Mutation status RET/HRAS | TNM | Calcitonin (pg/ml) | Analysis Pre-surgery | Analysis at follow up (m - y) | status at last follow up | type of analysis (F/M) |
| --- | --- | --- | --- | --- | --- | --- | --- | --- | --- |
| III-1-2 | F | 36 | RET | T1 N1aM0 | 42,4 | yes | 3 m | DF | F, M |
| III-15-1 | F | 61 | KRAS | T1bN0M0 | 525 | yes | 3 m | DF | F, M |
| I-88-1 | M | 75 | WT | T2N1bM1 | 1992 | yes | | deceased | F, M |
| I-107-1 | M | 72 | HRAS | T1bN0M0 | 1322 | yes | 3 m | DF | F, M |
| II-26-T0 | F | 47 | HRAS | T1aN1aM0 | 17,2 | yes | | DF | F |
| III-20-1 | M | 35 | RET | T1bN1bM0 | 15600 | yes | | BD | F, M |
| IV-1-1 | F | 45 | PIK3CA | T1N1M1 | NA | yes | | deceased | F, M |
| IV-11-1 | M | 76 | NA | T3N1MX | 12540 | yes | | deceased | F, M |
| III-14-1 | M | 70 | HRAS | T1AN0M0 | 90,7 | yes | 3 m | DF | M |
| III-18-1 | F | 38 | NA | T3N1bM0 | 3230 | yes | | deceased | M |
| I-61-1 | F | 54 | RET | T1bN0M0 | 4 | no | >5 y | DF | F |
| I-64-1 | M | 75 | RET WT | T2N1aM0 | 40 | no | 13 y | BD | F |
| I-65-2 | M | 46 | RET WT | T3aN1bM0 | 1948 | no | 10 y | SD | F |
| I-87-1 | F | 53 | RET WT | T2N1bM0 | 1526 | no | 22 y | BD | F |
| I-92-1 | F | 69 | RET | TxmN1bM0 | 334 | no | 14 y | BD | F |
| I-94-1 | F | 47 | RET WT | T3N1bM0 | 1450 | no | 13 y | SD | F |
| III-2-3 | F | 69 | RET WT | T1N0M0 | NA | no | 2 y | DF | F |
| III-4-3 | M | 44 | RET WT | T1N0M0 | NA | no | 2 y | DF | F |
| III-5-3 | M | 45 | RET WT | T1N0M0 | NA | no | 1 y | BD | F |
| III-8-1 | F | 50 | NA | T1N0M0 | NA | no | 2 y | DF | F |
| DF: disease-free |
| --- |
| BD: biochemical disease |
| SD: structural disease |
| |
| F= Fragmentation |
| M= Methylation |

## Slide 4
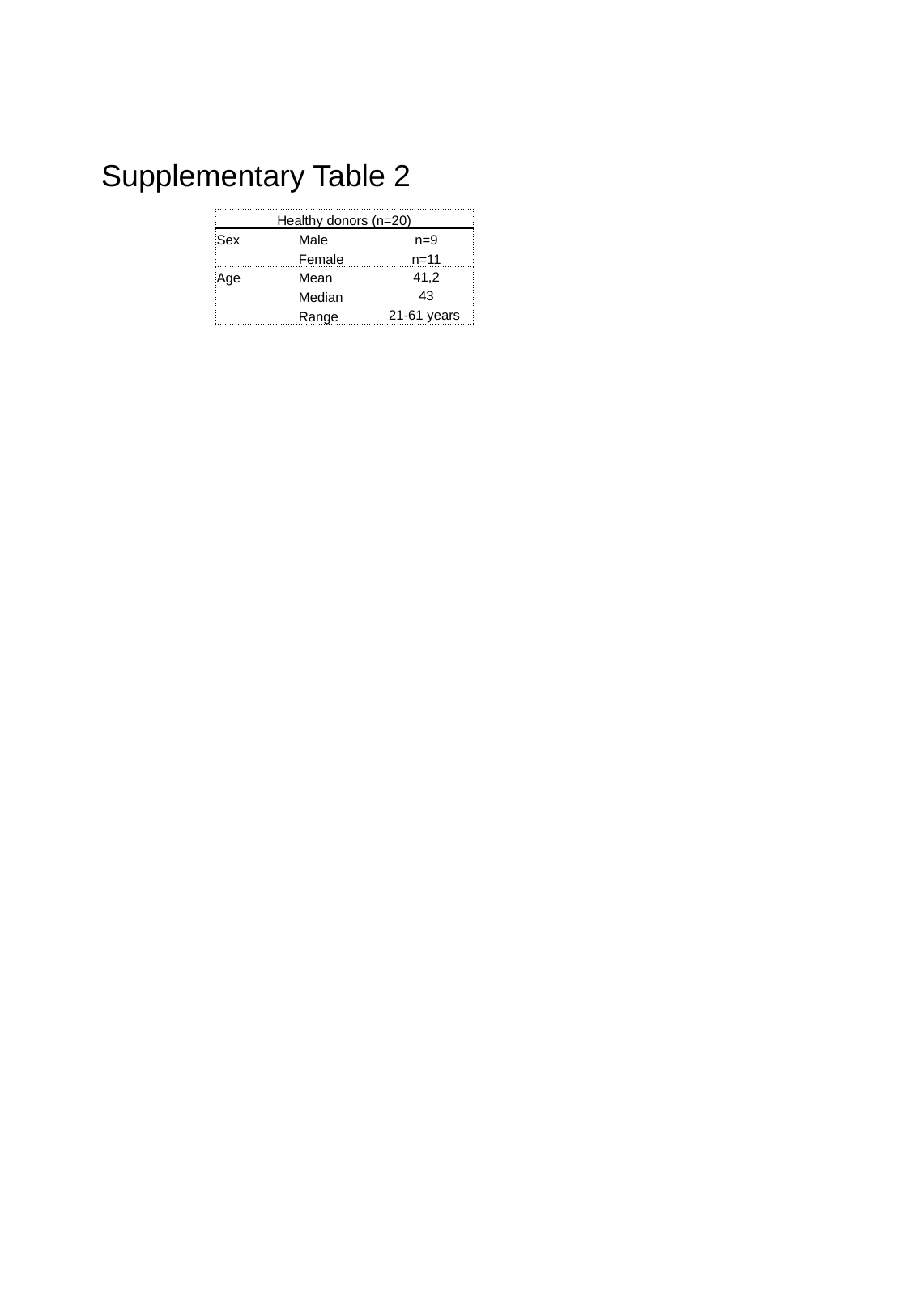

Supplementary Table 2
| Healthy donors (n=20) | | |
| --- | --- | --- |
| Sex | Male | n=9 |
| | Female | n=11 |
| Age | Mean | 41,2 |
| | Median | 43 |
| | Range | 21-61 years |

## Slide 5
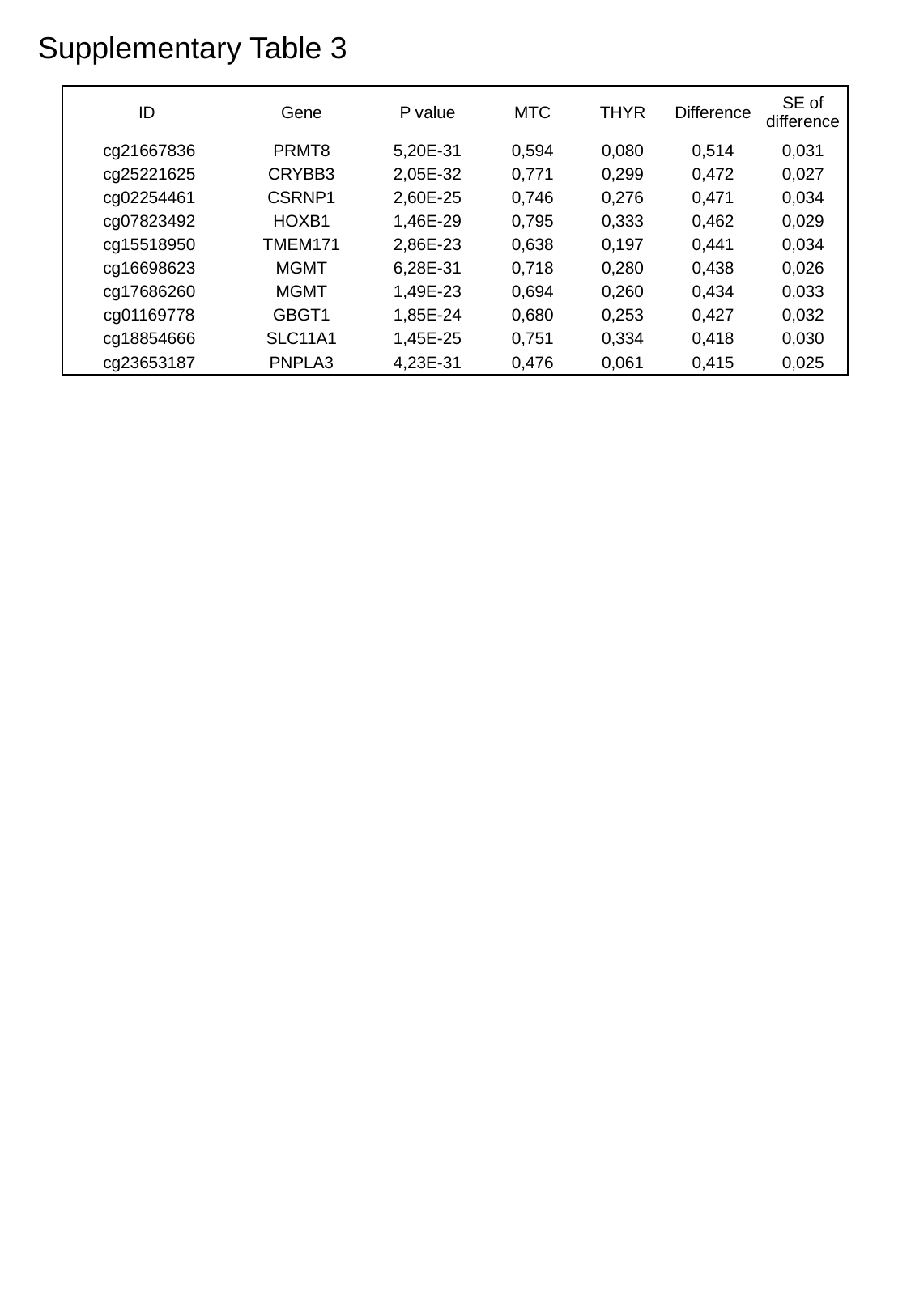

Supplementary Table 3
| ID | Gene | P value | MTC | THYR | Difference | SE of difference |
| --- | --- | --- | --- | --- | --- | --- |
| cg21667836 | PRMT8 | 5,20E-31 | 0,594 | 0,080 | 0,514 | 0,031 |
| cg25221625 | CRYBB3 | 2,05E-32 | 0,771 | 0,299 | 0,472 | 0,027 |
| cg02254461 | CSRNP1 | 2,60E-25 | 0,746 | 0,276 | 0,471 | 0,034 |
| cg07823492 | HOXB1 | 1,46E-29 | 0,795 | 0,333 | 0,462 | 0,029 |
| cg15518950 | TMEM171 | 2,86E-23 | 0,638 | 0,197 | 0,441 | 0,034 |
| cg16698623 | MGMT | 6,28E-31 | 0,718 | 0,280 | 0,438 | 0,026 |
| cg17686260 | MGMT | 1,49E-23 | 0,694 | 0,260 | 0,434 | 0,033 |
| cg01169778 | GBGT1 | 1,85E-24 | 0,680 | 0,253 | 0,427 | 0,032 |
| cg18854666 | SLC11A1 | 1,45E-25 | 0,751 | 0,334 | 0,418 | 0,030 |
| cg23653187 | PNPLA3 | 4,23E-31 | 0,476 | 0,061 | 0,415 | 0,025 |

## Slide 6
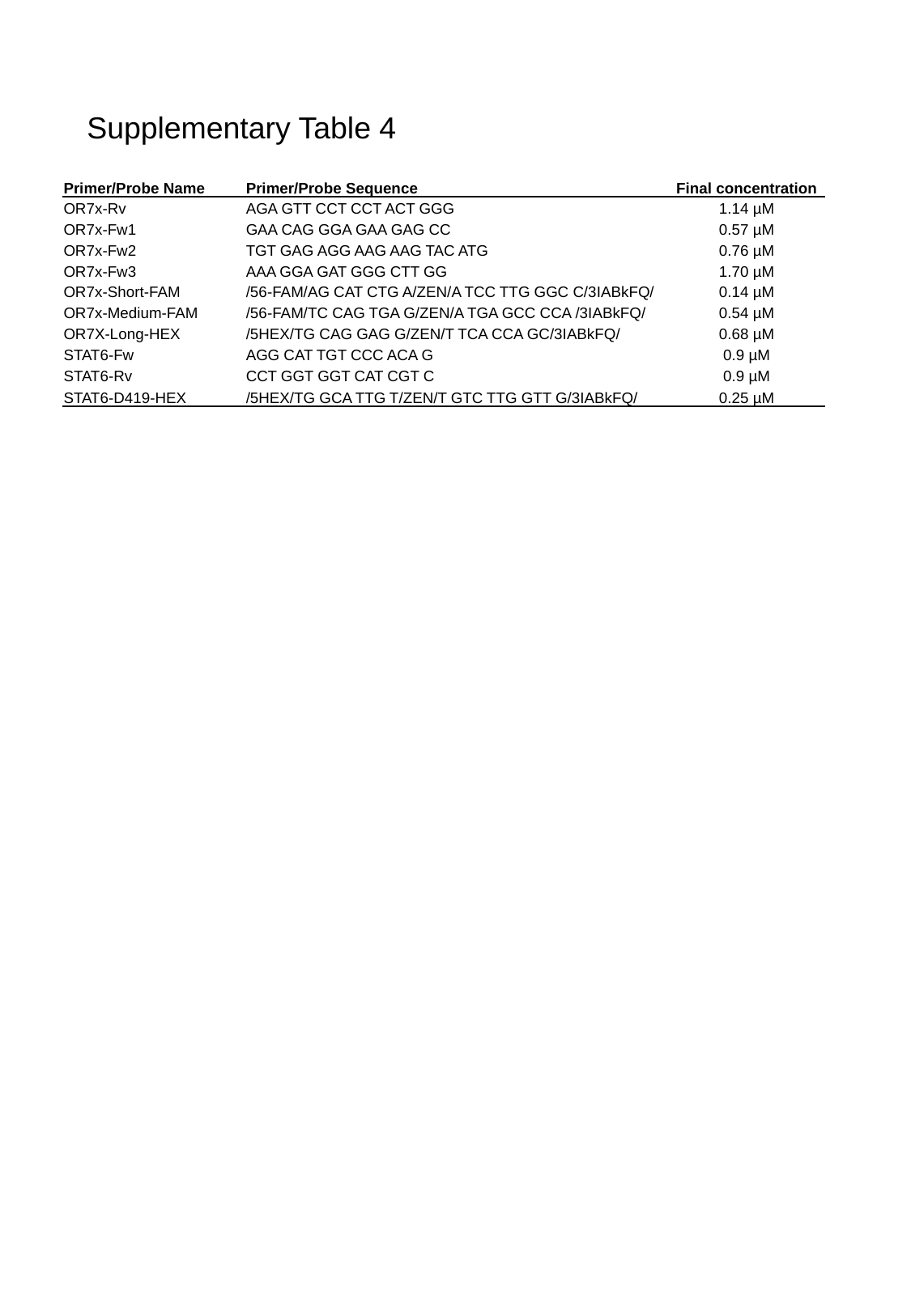

Supplementary Table 4
| Primer/Probe Name | Primer/Probe Sequence | Final concentration |
| --- | --- | --- |
| OR7x-Rv | AGA GTT CCT CCT ACT GGG | 1.14 µM |
| OR7x-Fw1 | GAA CAG GGA GAA GAG CC | 0.57 µM |
| OR7x-Fw2 | TGT GAG AGG AAG AAG TAC ATG | 0.76 µM |
| OR7x-Fw3 | AAA GGA GAT GGG CTT GG | 1.70 µM |
| OR7x-Short-FAM | /56-FAM/AG CAT CTG A/ZEN/A TCC TTG GGC C/3IABkFQ/ | 0.14 µM |
| OR7x-Medium-FAM | /56-FAM/TC CAG TGA G/ZEN/A TGA GCC CCA /3IABkFQ/ | 0.54 µM |
| OR7X-Long-HEX | /5HEX/TG CAG GAG G/ZEN/T TCA CCA GC/3IABkFQ/ | 0.68 µM |
| STAT6-Fw | AGG CAT TGT CCC ACA G | 0.9 µM |
| STAT6-Rv | CCT GGT GGT CAT CGT C | 0.9 µM |
| STAT6-D419-HEX | /5HEX/TG GCA TTG T/ZEN/T GTC TTG GTT G/3IABkFQ/ | 0.25 µM |

## Slide 7
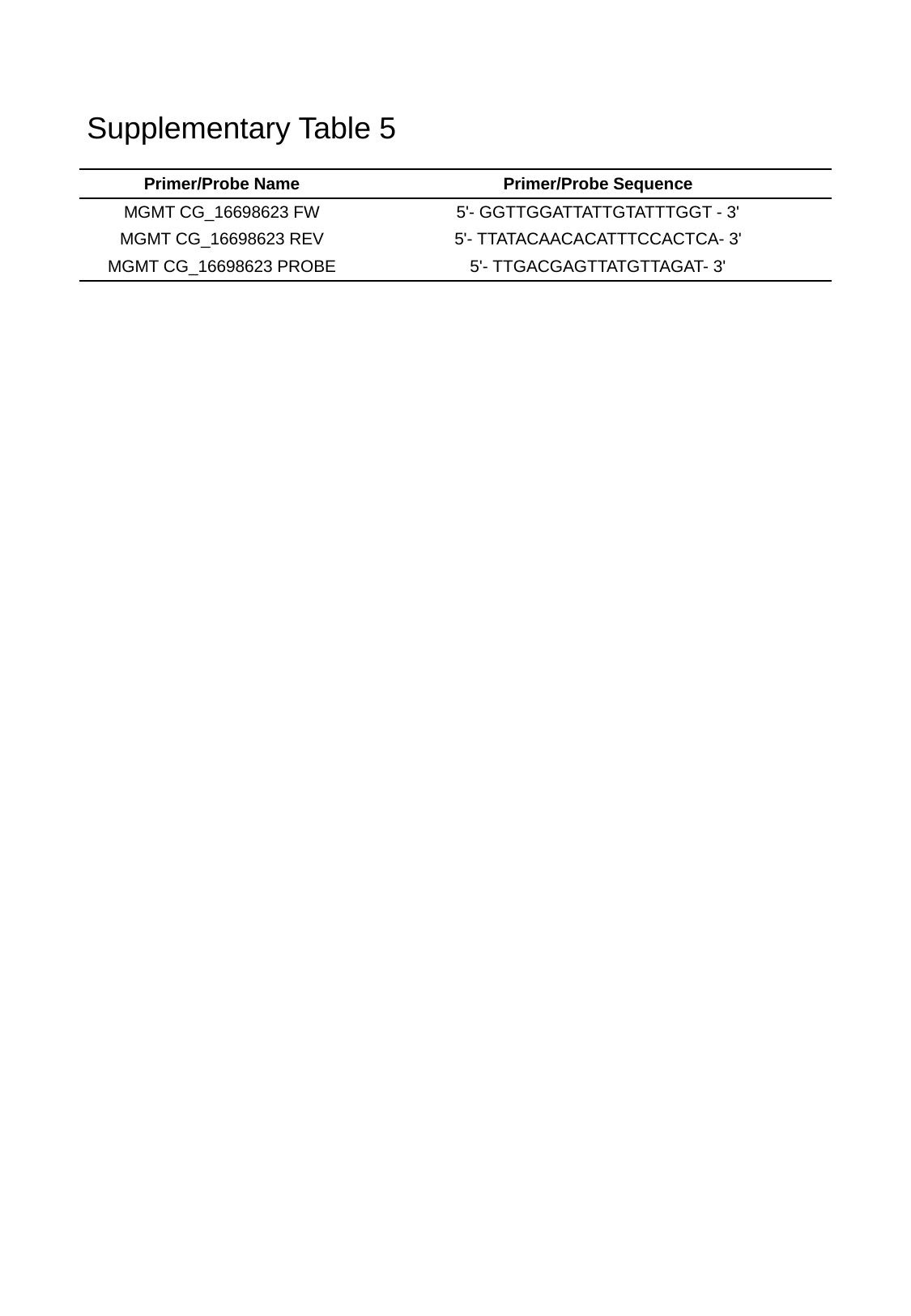

Supplementary Table 5
| Primer/Probe Name | Primer/Probe Sequence |
| --- | --- |
| MGMT CG\_16698623 FW | 5'- GGTTGGATTATTGTATTTGGT - 3' |
| MGMT CG\_16698623 REV | 5'- TTATACAACACATTTCCACTCA- 3' |
| MGMT CG\_16698623 PROBE | 5'- TTGACGAGTTATGTTAGAT- 3' |
